# Learning from an experience, challenges and approaches in the workplace during COVID-19 pandemic: a content analysis of international documents

**DOI:** 10.1186/s12889-024-19251-w

**Published:** 2024-07-09

**Authors:** Masoud Motalebi Ghayen, Mitra Faghihi, Elahe Ezati, Yahya Khosravi, Ali Almasi, Ali Asghar Farshad, Narmin Hassanzadeh-Rangi, Shayesteh Shirzadi

**Affiliations:** 1 Occupational Health Research Center, Iran University of Medical Sciences, Tehran, Iran; 2 Department of Public Health, School of Allied Medical Sciences, Asadabad Faculty of Medical Sciences, Asadabad, Iran; 3 Department of Occupational Health and Safety Engineering, School of Health, Alborz University of Medical Sciences, Karaj, Iran; 4 Non-Communicable Diseases Research Center, Alborz University of Medical Sciences, Karaj, Iran; 5 Social Development & Health Promotion Research Center, Health Institute, Kermanshah University of Medical Sciences, Kermanshah, Iran; 6 Department of Occupational Health Engineering, School of Public Health, Iran University of Medical Sciences, Tehran, Iran; 7 Research Center for Health, Safety, and Environment, Alborz University of Medical Sciences, Karaj, Iran; 8 Healthy Ageing Research Centre, Neyshabur University of Medical Sciences, Neyshabur, Iran; 9 Department of Public Health , Neyshabur University of Medical Sciences, Neyshabur, Iran

**Keywords:** Approach, Challenge, COVID-19, International guideline, Literature review, Lesson learned, Workplace

## Abstract

**Background:**

This study aims to investigate international measures for pandemic control in the workplace based on guidelines from international organizations to learn from their experiences.

**Methods:**

We conducted a qualitative study using content analysis. The search method involved reviewing published guidelines on preventing and responding to the COVID-19 pandemic in workplaces. After the screening process, ten guidelines were included in the content analysis. During the analysis, 200 meaning codes, 49 subcategories, and eleven categories were identified. Trustworthiness criteria were utilized to ensure the accuracy and strength of the findings.

**Results:**

Eleven categories of international content during the COVID-19 pandemic were legal requirements and duties of employees and employers, structural and program changes, risk assessment, risk communication, information and training, internal and external consultation and cooperation, provision of facilities and tools for workplace hygiene, special conditions, special groups, closing and reopening workplaces, reducing contact and exposure and mental health.

**Conclusions:**

Protecting employees during a pandemic requires a multifaceted approach and strong advocacy. The operational plan for pandemic control should be developed based on the level of risk, with support tailored to employees’ conditions and needs. Cooperation among international organizations is essential to develop a standardized plan and issue comprehensive guidelines in response to health emergencies with a global perspective and local implementation, drawing from the lessons learned during the COVID-19 pandemic.

## Background

On January 30, 2020, the World Health Organization (WHO) declared the outbreak of COVID-19 as a public health emergency of international concern [1]. The sudden appearance of COVID-19 affected people’s lives and the economy [2], and the world has suffered a lot from this disease in terms of human lives, economy, and increase in poverty [3]. The COVID-19 pandemic is both a global health crisis and an international economic threat [4]. New epidemics create severe stress that is widespread and uncontrollable compared to the stress experienced in everyday life [5].

Work is a significant risk factor in the transmission of infection [6]. In a study conducted in 6 Asian countries, in addition to healthcare workers, drivers, and transportation staff, service and sales workers, cleaning and domestic workers, and public safety workers accounted for the most cases of infection [7]. In another study, healthcare staff, emergency response workers, and social service and elder care workers were at the highest level of occupational risk. Conditions such as overcrowding, heavy transportation and traffic, multiple high-contact areas, and problems related to the impossibility of physical distancing in the workplace can lead to a high risk for SARS-CoV-2 transmission [7, 8].

On the one hand, workplaces play a role in the transmission and spread of infection due to task variety such as communicating with customers, patients, and colleagues, preparing food, etc. On the other hand, Public, business, and industry shutdowns to control the spread of the virus create a wide range of unique challenges for employees and employers, and the public health crisis of COVID-19 is quickly turning into an economic crisis [4]. The shutdown of businesses such as the tourism industry and food preparation and distribution centers has caused an economic burden on the communities [9]. The COVID-19 pandemic has created a lot of uncertainty and has significantly affected the supply chain, consumption, production, operations, valuations, security, financial stress, and the price of all products [10].

In Italy, 30% of working-age people (15–65 years old) were infected at the workplace [11]. In a study conducted in Canada, individuals who were close to patients, and workers who worked closely with co-workers were significantly more likely to develop a severe form of the disease. The mental health of the most affected workers during the pandemic was also significantly lower [12]. Working conditions also impact the mortality rate from COVID-19, with jobs involving contact with patients and the general public showing significantly higher [13].

At the onset of the pandemic, very little was known about the virus, including its mode of transmission, incubation period, medication and treatment, vaccination, and other related factors. Initially, the global community was not overly concerned, as it was believed that only those in contact with the Wuhan seafood market were at risk of infection [14]. Consequently, measures such as the closure of businesses related to this area the cessation of food sampling, and the prohibition of the sale of live and wild animals to restaurants and markets were implemented. Additionally, measures related to environmental health and market disinfection were carried out. Also, measures were taken to increase public awareness, especially for those who raised these animals [15, 16]. After it was suggested that the virus could be transmitted from person to person as a respiratory pathogen [16], China’s neighboring countries increased their health precautions. Japan began to establish comprehensive screening for travelers coming from Wuhan, and people with fever or flu symptoms were quarantined. In the United States, the Center for Disease Control and Prevention (CDC) created a 2019-nCoV incident management structure to prepare for future cases [17]. In general, following these uncertainties, numerous policies were issued, some of which were rendered ineffective as more knowledge about the virus emerged. For example, at first, the WHO recommended the use of masks only for health professionals or patients in hospitals. However after the possibility of human-to-human transmission was determined, it was urged that to prevent transmission or infection of the virus, everyone should wear a mask in public places [14]. In this regard, several recommendations and guidelines were published by organizations and countries, regarding preventive measures against COVID-19 in the workplace. Some of these include training employees, sending sick employees home, facilitating teleworking, reducing unnecessary trips, and avoiding social gatherings [18]. These measures resulted in beneficial outcomes, such as the implementation of physical distancing rules in the workplace being associated with a reduction in the physical absence of employees [19] and a decrease in infections among employees. This, in return, led to a reduction in the number of employee health insurance claims, improved morale, and provided more benefits for employers [20].

COVID-19 has taught us a lot of things we didn’t know, and we need to provide them in the future. It also reminded us of lessons and guidance that had been neglected and sidelined, possibly leading to preventable employee illnesses and deaths. Due to the risk of future epidemics, workplaces must strengthen their capacity, healthcare facilities must be reviewed and upgraded on time to control the outbreak without endangering the safety of employees. Occupational health services and hygiene measures, including barriers, ventilation, and personal protective equipment, will be needed on a much greater scale than before the start of the pandemic. Therefore, vigilance, protection, preparation, and subsequent planning for dealing with such incidents in workplaces are necessary, and a lack of prior investment will result in high costs [21]. Therefore, comprehensive guidelines are necessary to prevent and control COVID-19 and similar pandemics in the workplace. These guidelines provide a framework for creating a safe working environment for employees [22]. They outline the responsibilities of both employers and employees, as well as the necessary changes in workplace structure and programs [23]. Additionally, the guidelines address the provision of facilities and tools, workplace hygiene, and the need for special measures for certain groups [24]. Studies have shown that implementing comprehensive infection prevention and control measures can effectively reduce workplace transmission [25]. Therefore, these guidelines serve as a valuable resource for employers and employees in preventing the spread of COVID-19 and similar pandemics in the workplace. It is necessary to learn from the events and measures taken to control the disease in workplaces. One way is to review the documents prepared by responsible international organizations. In this article, the control and prevention measures at the workplace based on the documents and guidelines of international organizations have been discussed. The present study aims to investigate international measures of pandemic control at the workplace based on the guidelines of international organizations and learn from them and their experiences.

## Methods

**Fig. 1:**
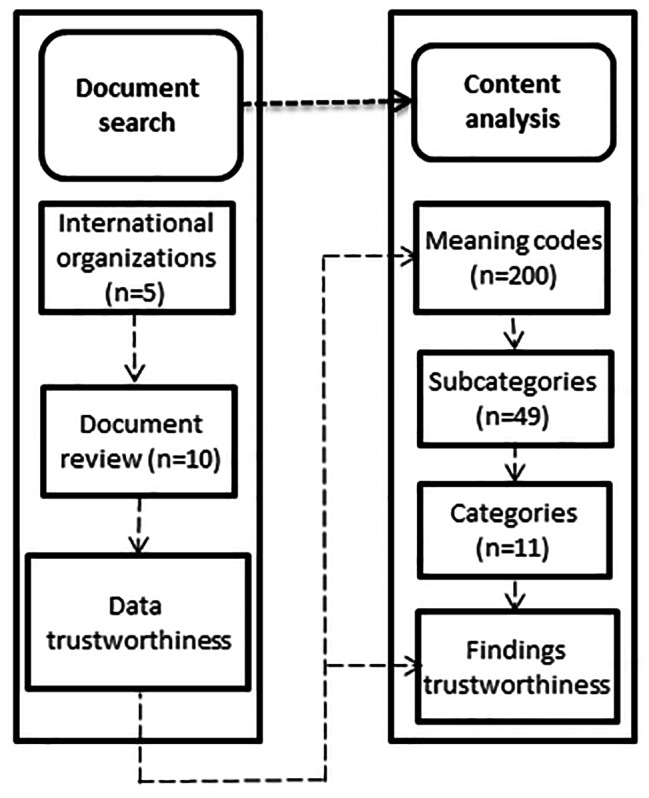
Study flow diagram

This study is part of a large project aimed at explaining the implementation model of prevention and emergency response to the COVID-19 pandemic and other acute respiratory infections in Iranian workplaces [26–28]. Figure 1 illustrates the study flow diagram.

The search for information was conducted based on guidelines published by international organizations such as WHO [18, 29, 30], the International Labor Organization (ILO) [31], American Occupational Safety and Health Administration (OSHA) [32, 33], CDC [34–36], European Agency for Safety and Health at Work (EU. OSHA) [37]. After the screening process, ten guidelines were included in the study. The review focused on extracting information about required and recommended changes in the workplace and working methods. Measures taken to address these conditions and reduce the threats were also examined. Data analysis was conducted using the content analysis method. To achieve this, all documents were carefully read multiple times by a member of the research team. The analysis began with identifying semantic units, generating codes inductively, and categorizing them into different classes and sub-classes based on differences and similarities. During the analysis, approximately 200 meaning codes, 49 subcategories, and 11 categories were identified. This study utilized the criteria proposed by Guba and Lincoln to ensure the accuracy of the study. Guba and Lincoln outlined four important principles to assess the accuracy and strength of qualitative studies [38], including credibility, confirmability, transferability, and dependability. To enhance the likelihood of obtaining genuine and valid findings, the researchers engaged with the subject under study for an extended period. The extracted texts were thoroughly studied multiple times both generally and in detail by the researcher. Observer review was another method employed to validate the data. The text of the interviews along with the extracted codes and categories, was reviewed by another member of the research team to verify the accuracy of the coding process. Additionally, all extracted codes were reviewed by a member of the research team, and external experts also reviewed the data to ensure validity. During the process, codes, text excerpts, and subclasses were reviewed by experts at the beginning and end of the study. Feedback from these experts was incorporated to enhance the quality of the study. To ensure the reliability of the data, the researcher shared the findings with other researchers and qualitative experts to validate the conclusions reached. Any discrepancies were addressed through further review and revision of codes and classes. To ensure verifiability, the translated texts were reviewed by supervisors. The researcher also aimed to enhance the transferability of the findings by interpreting, analyzing, and describing the data in a way that allows for evaluation and comparison with other studies.

## Results

Based on the results of the document review on prevention and response to the COVID-19 pandemic in workplaces, a total of 11 main categories and 49 subcategories were identified. The main categories include legal requirements and duties of employees and employers, structural and program changes, risk assessment, risk communication, information and training, internal and external consultation and cooperation, provision of facilities and tools for workplace health, special conditions, special groups, closing and reopening workplaces, reducing contact and exposure and mental health (Table 1).

### Legal requirements and duties of employees and employers

The legal requirements and duties of employees and employers that should be taken into account include:

1. Cooperation and compliance of employees and employers with infection control and prevention programs and measures, such as compliance and implementation of protocols issued by relevant health authorities and organizations such as the labor union, local laws and regulations, participation in training provided by the employer and relevant authorities, immediate reporting of any imminent and serious health risk situation to the supervisor, absence from the workplace and participation in meetings if having symptoms and infection, notifying health care centers in case of symptoms and staff infection, being aware of the latest developments and recommendations [30–32, 36].
2. Frequent monitoring and supervision of compliance with regulations, implementing protocols, and updating them, such as updating operational plans and control measures in response to changes in the epidemiological trend, are essential. These include monitoring and supervising the implementation of preventive and protective measures like distancing rules, personal hygiene, environmental hygiene, and surface disinfection [29, 31, 34].
3. Providing the facilities and taking protective and preventive measures by the employers [30, 31, 36].
4. Respecting the rights and duties of employees and employers in occupational safety and health, such as rules and agreements on working hours in conditions of increased workload, prioritizing the health and safety of employees and keeping work pressure low, and the confidentiality of any details about the medical condition of employees [31, 32, 36, 37].
5. Lack of anti-retaliatory measures against employees who follow the rules and instructions [34].

### Structure and program changes

Based on the study results, changes in workplace structure and programs are necessary during the COVID-19 pandemic. These measures include:

1. Workforce management, such as identifying multi-skilled employees, having a plan for how employees work in conditions of reduced staff, changing roles and responsibilities, providing necessary training and support, hiring temporary employees, shifting and dividing work, and identifying employees with IT skills [18, 35–37].
2. Flexible work policies such as remote working, leave, reduction of working hours, relocation of activity, change in work organization or work duties of vulnerable people or people with special conditions, permission to be absent at the workplace, even in case of mild symptoms [18, 29, 30, 33, 34, 36, 37].
3. Designing an action plan related to observing a person with suspicious symp toms and a sick person at the workplace, including limiting the number of people in contact with the sick person, identifying people at risk and supporting them without introducing stigma and discrimination, reviewing and confirming communication and information channels in advance with key partners such as public health and health care authorities, preparing a plan for how to safely transfer them to health centers, follow up on people in contact with a person with symptoms and a patient, counseling remote medicine or canceling the need for a medical note for people to stay at home, if possible, isolation room [18, 29–31, 33, 34, 36].
4. Self-assessment and symptom screening through questionnaires, measuring body temperature for employees, customers, and clients [29–31, 33, 34, 37].
5. Considering care and accommodation centers including, equipping children to care centers at work, considering accommodation centers for employees with special conditions [32–34].
6. Travel management including evaluating benefits and risks based on the latest information, canceling and limiting travel, monitoring and controlling symptoms before and after travel, follow-up, and quarantine, being informed of the latest information related to outbreak areas, avoiding sending people at risk, providing necessary equipment and materials, notifying health centers about people who have traveled to outbreak areas (before and after returning), complying with Local authorities’ instructions, or local restrictions related to travel [18, 29, 33–35].
7. Drafting a contingency plan and continuity of trade/business in the conditions of the outbreak of COVID-19 and financial support, such as having a plan and considering services for contract or temporary employees, designing a plan with social and health service providers for small and medium businesses without welfare and health support of employees, paying minimum wage for people suspended from work for a longer period and suspected people required to be quarantined or positive people, adjustment subsidy for vulnerable jobs, paying compensation to employers supporting employees and caring for vulnerable people, online orders and remote service provision business continuity plan and employee activities in case of a significant reduction in the number of employees, contractors, and operators [18, 35, 36].
8. Setting up workstations at home and workplace such as IT equipment, software, and good ergonomics [35, 37].

### Risk assessment

Risk assessment is one of the actions and strategies that can have a significant impact on how to function and develop plans and actions for disease prevention and control in business locations. The things that should be taken into consideration in the risk assessment are:

1. The work environment and working group in terms of the amount of exposure and contact with the public, colleagues, clients, and customers, contact with people infected or suspected of COVID-19, population density, contact with objects or surfaces infected with the virus [29, 31, 33–35].
2. The epidemiological status of the disease [34, 35, 37].
3. The level of vulnerability of people including personal and medical conditions [18, 29, 34, 37].
4. Physical and psycho-social risk assessment [18, 36, 37].

### Information, education, and risk communication

In the conditions of a pandemic like COVID-19, it is necessary to consider education and information as the main pillars. The things that should be considered are:

1. Providing general education about the disease and training for employees with symptoms and infected individuals, covering symptoms, prevention methods, medications to manage symptoms, self-treatment, etc. [18, 29–31, 33–36].
2. Training related to groups and special conditions, including vulnerable people, employees of special departments such as the cleaning department, managers, employers, and line managers, marginal groups such as workers in the Informal economy and migrant workers, domestic workers, secondary and self-employed workers, training related to changing roles and responsibilities, use of equipment and software needed in remote working conditions, digital platforms, providing remote and online services, setting up a workstation with proper ergonomics and frequent movement, other occupational health and safety risks such as ergonomic problems, heavy loads and long working hours, remote working, psycho-social risks, poisoning, coping with grief and., how to access correct information and identify reliable sources [29, 31, 33, 35–37].
3. Training-related instructions, including the use of personal protective equipment, disinfectants, travel management (such as maintaining hygiene and physical distance, etc.), being informed about actions and the people who should be contacted in case of feeling sick, instructions from local authorities), management of meetings, principles of ventilation [18, 29–31, 34, 35, 37].
4. Training related to reducing restrictions, restarting, and returning to work should include informing and training employees on procedures and plans for returning to work and the latest developments, recommendations, and changes [31, 36, 37].
5. Planning communication programs and using the capacity of different communication channels, such as determining and introducing a single and reliable source of information and response in the organization. Reviewing and identifying communication channels with health authorities, informing about the actions taken, and processes, and providing feedback about preventive measures and their effectiveness. Neutralization of false information and rumors, availability, support, and awareness and attention to the concerns of employees, informing about the latest developments and recommendations, selecting, creating, and using the capacity of different communication channels for training, informing and reminding, and installing them in the right place, including posters, videos and electronic message boards, campaigns, infographics, internet-based communications, etc. [18, 29, 31, 33, 35–37].

### Internal and external consultation and cooperation

The collective study takes place through participation, consultation, and communication with the following sectors and in designing programs and carrying out control and prevention measures such as risk assessment, return to work, etc. among other things that should be considered.

1. Employees or their representatives [29–31, 35, 37].
2. Providers of occupational health services, risk prevention, and health and safety consultants [31, 36, 37].
3. Local officials, organizations, and businesses at the local level and labor unions [31, 35].
4. Authorities and healthcare system [18, 31, 35].
5. National travel advisor [18, 35].
6. Insurance companies and state and local health agencies [33, 36].

### Provision of facilities and tools and workplace hygiene

Workplace hygiene and the provision of facilities and equipment are other necessary measures to control conditions similar to the COVID-19 pandemic. These actions include:

1. Compilation of the workplace hygiene plan, including preparation of daily cleaning protocol, maintenance and display of records of cleaning and disinfection activities, allocation of sufficient time and manpower for cleaning and disinfection, identification of areas and surfaces with high traffic and a lot of touches to pay special attention to them (dining room, closet/dressing room, corridors, door, and window handles, counters, handrails, light switches, handles, elevator door buttons, commuting services, workstations, isolation room, toilet doors, etc.), compliance with the principles of cleaning and sanitizing the workplace (the sequence of cleaning and disinfection, based on open and closed workplaces, the type of surfaces and equipment, choosing disinfectants under the regulations of local authorities market approval opinion, preparation, and use of disinfectants according to instructions, proper and hygienic disposal of equipment) [18, 29, 31, 34, 35].
2. Maintaining and promoting personal hygiene such as thorough, regular, and frequent hand washing according to instructions, especially after contact with secretions, body excrement, and potentially contaminated objects (gloves, clothes, masks, used napkins, scraps) and immediately after removing gloves and other protective equipment, creating hand hygiene stations and placing them in the right place (for employees, clients or customers, etc.), respiratory etiquette, formulating a policy of wearing a mask or covering the face according to the instructions [18, 29–31, 33–35, 37].
3. Improving air hygiene, including increasing natural and artificial ventilation, preferably without air recirculation, specialized negative pressure ventilation in some places (aerosol production procedures), the openness of work environments, and installation of high-efficiency air filters [18, 29, 31, 33].
4. provision of equipment related to personal protection and workplace hygiene (such as masks, disposable gloves, shields, disinfectants, no-touch trash bins, equipment needed by cleaning staff, etc.) [18, 29–31, 33, 35, 37].

### Special conditions

In pandemics similar to COVID-19, special conditions are created and measures must be planned to adjust and deal with them. These conditions are:

1. Reduction in the number of employees and increased absenteeism due to reasons such as illness, caring for sick family members and children, the presence of at-risk individuals in the family, fear of infection, death of relatives, or unwillingness to return to the workplace after reopening [18, 33–35, 37].
2. Increasing workload [29, 37].
3. Suffering from other problems and diseases such as working diseases caused by poisoning, ergonomic problems mental health problems [29, 33].
4. Financial problems and changes in the business model due to the increase in demand for items related to infection prevention and disease control and the decrease in consumer interest in other goods, shopping during off-peak hours, interest in home delivery services, job insecurity, sudden loss of income, delay or cancellation with or without notice of shipping items from geographical areas with high prevalence or due to travel restrictions [18, 33–35].
5. According to the results of the study, one of the things that should be considered in crises similar to the COVID-19 pandemic is to pay attention to employees with special conditions. These are people who are more vulnerable due to personal conditions, or people who are more vulnerable due to the emerging pandemic that needs special attention, including:

### Special groups

1. vulnerable people due to individual and medical conditions such as old age, underlying and chronic disease, pregnancy, etc. [18, 29, 31, 33–35, 37].
2. People with special conditions, including having a baby or a young or school-aged child, conditions of employees’ spouses, having a vulnerable family member, the existence of the affected and isolated person in the family, and employees who have lost their relatives [31, 33–35, 37].
3. People involved with the disease include employees with the disease, people with a severe form of the disease, and employees after returning to work (individual isolation) [31, 34, 36, 37].
4. People with financial problems such as temporary and contract employees, and people with reduced or lost income [18, 29, 31, 33].
5. Remote workers, This group of people requires special attention and their conditions should be considered in planning and decision-making [18, 29, 31, 36, 37].

### Closing and reopening workplaces

One of the key considerations during a pandemic like COVID-19 is designing a return-to-work plan and paying attention to the considerations of returning to work. These items include:

1. Determining criteria, limits, and prioritization in returning to work, in terms of employees, groups, departments or units, exposure level, etc., according to business needs, determining criteria and limits for reduction of COVID-19 to allow the resumption of work activities, the capacity to implement preventive measures and the recommendations of national authorities to regulate social and public health measures [18, 29, 31, 34–37].
2. Preparation for returning to work includes distancing and reducing density, disinfection, cleaning, and preparing equipment, etc., informing employees of changes, procedures, and plans before starting work, updating risk assessments, considering ways to communicate and respond to questions or concerns, providing relevant training to return to work and considering measures to respond to any emergence or resurgence of COVID-19 in the workplace and community [31, 36, 37].
3. Paying attention to the low threat of considering the conditions after returning to work and repeatedly emphasizing and monitoring the requirements of implementing preventive measures, is a protection of things that should be considered in planning and taking action to return to work [31].

### Reduction of contact and exposure

Reducing contact and exposure in the workplace is one of the basic measures to control and prevent disease in the workplace, these measures include:

1. Physical changes in the workplace such as marking the floor, blocking, using an electronic system and a personal card when entering and exiting, reducing the number of areas with a lot of touches or high traffic (for example, leaving some internal doors open, facilities related to opening and closing doors without contact), not using common items in the reception, dining areas, etc., putting employees next to each other instead of face-to-face [18, 29, 31, 33–35, 37],
2. Changing the way of providing services to customers and clients, including eliminating or reducing face-to-face contact and physical interaction (online orders, installation of a stimulus portal for customer service, delivery outside the workplace, remote service provisions such as telephone or video), reducing contact time in cases where close contact is unavoidable, managed entry and exit and limiting access to customers and the general public to the workplace (determining the maximum number of people who can stay inside the workplace at the same time, limiting the entrances and entering the workplace if necessary, attending with setting an appointment in advance) [31, 33–35, 37].
3. Administrative-executive controls to reduce the density of people and to limit the capacity, reducing, working hours and alternating working hours, shift or team division arrangements, avoiding crowding when entering and exiting (through alternating shifts or intermittent entry and exit, an extension of exit time), work shift, postponing or suspending work events including close and long-term contact between participants and social gatherings, management of meetings and conferences by examining the need to be present and the possibility of replacement online or teleconference, safe distance, ventilation, reducing the number and time of meetings, provision of personal protective equipment, disinfectants, etc. Also, not allowing the presence of people with symptoms, receiving information of people present in the meeting for follow-up and necessary action in case of necessity, avoiding direct physical contact with other people (hugging, touching, shaking hands), avoiding crowding in workstations and common spaces (such as entrances/exits, corridors, elevators, cafeterias / diners / stores, stairs, gathering places or queues of employees or visitors/customers) through timing, spacing and reducing the frequency, reducing contact between different departments at the beginning and end of the work shift, managing mass transportation and complying with protocols (encouraging individual transportation, providing suitable and safe parking places and bicycles, encouraging walking to work if there is a place, complying with protocols public transportation) [18, 29, 31, 33–37].

### Mental health

Paying attention to the mental health of employees and its threatening factors, taking measures, and providing services to maintain and improve their mental health are other things that should be considered.

1. Some factors can affect and threaten people’s mental health. should be regarded: conditions such as the COVID-19 pandemic and the pressures caused by the epidemic, contracting a severe form of the disease, fear of contracting the disease and death, especially in vulnerable people or those with vulnerable family members, the death of relatives and friends, problems related to relationships personal, concerns after returning from isolation, whether individual or collective (risk of infection and job changes, etc.), furthermore, risk of feeling isolated and under pressure from workers, uncertainty and anxiety, fear of losing job or income, financial problems pressure on parents and caregivers of children (closure of schools and kindergartens), increase in workload demand, anxiety of drowning in information, stigma caused by illness, reduction of people’s social connections, long quarantines, remote working, loss of small businesses, uncertainty due to not knowing how long the crisis will last, etc. are all factors that can affect and threaten people’s mental health [18, 29, 31, 33, 35–37].
2. Measures that can be used in order to maintain and promote mental health in the conditions of an epidemic include assessing the mental health of employees, de-stigmatizing people with the disease, providing psycho-social support, supporting anxious and high-stress employees, facilitating exchange and socializing between colleagues, changing the organization of work or work tasks, contacting occupational health services, providing information about support resources, support and advice, establishing and strengthening communication at all available levels (from top management to line manager duties, without forgetting the importance of the usual social interaction among colleagues, establishing effective communication and support from the manager and colleagues and the ability to communicate informally with colleagues, establishing healthy boundaries between work and leisure, the availability of managers and answering questions, using doctors and health care staff for advice, respecting workers’ privacy and confidentiality, considering policies and guidelines for contract or temporary employees, being aware of and addressing employee concerns about pay, leave, safety and health, considering non-occupational risk factors at home and social environments, addressing needs and concerns in case remote working is not possible, considering the risks affecting mental health after resuming work and returning to work, limiting media consumption, compassionate leadership culture, equipping the line managers to the skills and tools necessary to investigate and recognize the signs of distress [18, 29–31, 33, 35, 37].

**Table 1:** Contents of international guidelines for controlling COVID-19 in the workplaces

Main categories	Subcategories
**Legal requirements and duties of employees and employers**	- Cooperation and compliance of employers, employees, and their organizations in the field of prevention and control of COVID-19 [30–32, 36] - Procurement, preparation, and supply of protective and preventive equipment and measures by employers [30, 31, 36] - Respecting the rights and duties of employees and employers stipulated in occupational safety and health [31, 32, 36, 37] - Absence of anti-retaliatory measures against employees [34] - Frequent monitoring and supervision of compliance with regulations and protocols and updating them [29, 31, 34]
**Structure and program changes**	- Workforce management [18, 35, 37] - Flexible work policies [18, 30, 33, 34, 36, 37] - Designing a program of measures related to the observation of a suspicious person, with symptoms and a patient [18, 29–31, 33, 34, 36] - Self-assessment and symptom screening [29–31, 33, 34, 37] - Considering care and residential centers [32–34] - Travel management [18, 29, 33–35] - Development of a contingency plan and continuation of trade/business in the conditions of the outbreak of COVID-19 and financial support [18, 35, 36] - Setting up workstations, equipment, and software [35, 37]
**Risk Assessment**	- Risk assessment based on work environment and occupational group [29, 31, 33–35] - Risk assessment based on individual and medical conditions [18, 29, 34, 37] - Identification and assessment of physical and psychological-social risk [18, 36, 37] - Epidemiology status of the disease [34, 35, 37]
**Information, education and risk communication**	- Public education about the disease and education of employees with symptoms and infected people [18, 29–31, 33–35] - Training related to special conditions and groups [29, 31, 33, 35–37] - Training-related instructions [18, 29–31, 34, 35, 37] - Training related to reducing restrictions and returning to work [31, 36, 37] - Developing communication plans and using the capacity of different communication channels [18, 29, 31, 33, 35–37]
**Internal and external consultation and cooperation**	- Employees or their representatives [29, 31, 35, 37] - Providers of occupational health services, risk prevention, and health and safety consultants [31, 36, 37] - Local officials, organizations, and businesses at the local level and labor unions [31, 35] - Authorities and healthcare system [18, 31, 35] - National travel advisor [18, 35] - Insurance companies and state and local health agencies [33, 36]
**Provision of facilities and tools and workplace hygiene**	- Compilation of workplace hygiene program [18, 29, 31, 34, 35] - Maintaining and promoting personal health [18, 29–31, 23, 25–28] - Maintaining and promoting air health [18, 29, 31, 33] - Provision of personal protective equipment and workplace hygiene [18, 29–31, 33, 35, 37]
**Special conditions**	- Increased absenteeism and reduced number of employees [18, 33–35, 37] - Financial problems and changes in the business model [18, 33–35] - Increasing workload demand [29, 37] - Suffering from other problems and diseases [29, 33]
**Special groups**	- vulnerable people [29, 31, 33–35, 37] - People with special conditions [31, 33–35, 37] - People involved with the disease [31, 34, 36, 37] - People with financial problems [18, 29, 31, 33] - Remote employees [18, 29, 31, 36, 37]
**Closing and reopening workplaces**	- Determining criteria, limits, and prioritization for returning to work [18, 29, 31, 34–37] - Preparing the workplace [31, 36, 37] - Considering the threat of being considered low-risk after returning to work [31]
**Reducing contact and exposure**	- Physical changes in the workplace [18, 29, 31, 33–35, 37] - Changing the way of providing services to customers and clients [31, 33–35, 37] - Administrative-executive controls [18, 31, 33–37]
**Mental health**	- Factors that threaten mental health [18, 29, 31, 33, 35–37] - Maintaining and promoting mental health [18, 29–31, 33, 35, 37]

.

## Discussion

Our goal in this study was to analyze the content of international guidelines related to the measures required to control COVID-19 in the workplace. Risk assessment and its updating are crucial strategies in formulating and implementing disease prevention and control programs in business locations [39]. In a pandemic like COVID-19, defining risk factors based on individual factors such as age, sex, chronic diseases, and factors related to the work environment such as exposure level and physical distance to create sensitivity to adverse outcomes to support public and occupational health policies is necessary [8]. Risk identification and assessment in the environment should be considered from all aspects, including physical and psychosocial [40]. Staff working in multiple care homes exhibited a risk of SARS-CoV-2 positivity that was 3.0 times higher than staff working in a single care home. Analysis of the entire genome sequence revealed separate groupings of SARS-CoV-2 infection among staff members exclusively, even among those who had minimal interaction with residents [41].

Identifying and assessing the physical and psychosocial risks of the work environment is the starting point of occupational health and safety management regarding measures related to COVID-19, which must be updated as conditions change. Risk assessment based on individual conditions, occupational groups, and work environment is one of the important measures in the control and management of epidemics and can be used as a guide for action such as the use of personal protective equipment and the type of equipment, measures and changes required in the work environment, training requirements as well as control-supervisory rules should be taken into consideration. There may be jobs with different levels of risk in similar work environments, and different jobs and work tasks may have similar levels of risk. Therefore, a risk assessment should be done for each specific work environment, job, or job group. For any risk assessment, the environment, task, and threat should be considered.

Based on the international documents reducing contact and exposure and providing the equipment needed for this purpose as well as tools and workplace hygiene were some of the important and priority measures to control COVID-19. Therefore to manage the spread of the disease in the workplace, control measures must be implemented to either eliminate or minimize the risk of exposure. Collective measures should be prioritized, supplemented by individual measures like personal protective equipment when necessary. Prevention and control of the risk of COVID-19 in the workplace in addition to the use of personal protective equipment requires structural changes, these structural changes include executive/administrative control such as work shift control, limiting capacity, practicing social distance, replacing face-to-face meetings with video conversations, conference etc., and change in physical structure and engineering control or separation of employees from work-related hazards such as physical barriers/separation shields, more ventilation of the workplace and… Also, safe work practices, which are a type of administrative control and include methods used to reduce the duration, frequency, or severity of exposure to hazards, such as providing resources and a work environment that promotes personal hygiene, for example, sanitary napkins, non-touch trash cans, soap, etc., installation of signs of hand washing in restrooms is another way to control infection in the workplace.

The results of a study showed after implementing both universal masking and physical barrier strategies, a statistically significant decrease in the occurrence of COVID-19 was observed in 8 out of 11 facilities within less than 10 days. In contrast, facilities that solely implemented a universal mask policy did not demonstrate any significant disparity in COVID-19 incidence rates pre- and post-intervention [42]. Based on the results of another study, the risk of COVID-19 infection was more than double among healthcare workers self-washing their masks compared with the hospital laundry. There was no significant difference in infection between healthcare workers who wore cloth masks washed in the hospital laundry compared with medical masks [43]. the results of a study in nursing homes showed changes in work arrangements and compartmentalization of staff within designated areas and the meticulous application of effective preventative measures can serve as a means to mitigate the occurrence of COVID-19 outbreaks [44]. Also, staff working in multiple care homes exhibited a risk of SARS-CoV-2 positivity that was three times higher than staff working in a single care home [41]. The results of a study showed that environmental monitoring of the coronavirus can help identify asymptomatic carriers and confirm the effectiveness of this measure in controlling COVID-19 in the workplace. The results of this study show that in places with a high prevalence of surfaces infected with the coronavirus, compared to places without contaminated surfaces or with low levels of contamination, the probability of having employees with a positive COVID-19 test result was 10 times higher. Therefore, with environmental monitoring, it is possible to predict the need for employee testing to identify asymptomatic cases, and environmental monitoring is a useful tool for managing the occurrence of COVID-19 in the workplace [45].

The National Institute for Occupational Safety and Health (NIOSH) has proposed a “hierarchy of control” in 5 levels from the most effective to the least effective, including removal, replacement, engineering controls, administrative controls, and personal protective equipment [40, 46].

Therefore, workplaces must define processes to identify risk factors as a guide for risk assessment and management procedures such as adopting preventive policies and measures and developing an action plan for disease control according to the level of risk. When choosing control measures, factors such as ease of implementation, effectiveness, cost, advantages, and disadvantages should all be taken into consideration. In most cases, a combination of control measures will be necessary to protect employees. The best way to control a hazard is to systematically remove it from the workplace, rather than relying on workers to reduce exposure. Engineering controls that involve isolating workers from work-related hazards, and inappropriate workplaces, reduce exposure to hazards without relying on worker behavior and can be the most cost-effective solution to implement. Administrative controls require action by employees or employers. Typically, administrative controls are changes in policy or procedures to reduce or minimize risk exposure.

Another thing that was mentioned in the documents is protecting the mental health of employees during the COVID-19 pandemic.

The results of a study showed that 3 months after the infection, female healthcare workers, older workers, and healthcare assistants were more likely to report persistent symptoms. Female workers perceived worse physical and mental health status at 3 months after the infection. Age had a negative correlation with physical health scores. The findings suggest that organizing specific interventions tailored to professional sub-groups can help protect and boost the physical and mental health of healthcare workers. The research provides insights into the long-term effects of COVID-19. The study underscores the importance of social support and positive working relationships in protecting mental health care workers.

Interventions to promote peer collaboration should be a priority for stakeholders [47]. The employee who returned to work after recovering from COVID-19 reported a need for programs centered around support, care, and stress management. Gender considerations should be taken into account when designing and implementing plans to help employees adapt to work. Women may be more likely to experience physical decline or fatigue, post-activity polypnea, and alopecia, and therefore, tailored support and modified work arrangements may be necessary. long-term follow-up visits should be implemented for infected healthcare workers to monitor their health condition, as pandemic survivors may suffer from long-lasting psychiatric symptoms and physical symptoms. Good organizational leadership, social support from co-workers and supervisors, and opportunities for employees to discuss and share their experiences with others who have had similar experiences can help in regaining normality in the workplace [48]. Providing mental and financial support to employee infected with COVID-19 during an outbreak period is important and can help reduce the turnover rate and encourage them to maintain their motivation to keep working [49].

Individuals with a severe form of the disease may require special attention even after returning to work. Issues that should be taken into account are muscle weakness, post-traumatic stress, memory and concentration problems, impaired task performance, poor problem-solving skills, failure to return to previous levels of performance, needing more time to resume activities, and suffering from stigma and discrimination. The closure of schools and kindergartens, as well as the circumstances of employees’ spouses and their employers, create challenging conditions for families. This necessitates the managers to implement flexible work policies and communicate their understanding and flexibility to employees. Additionally, if feasible, arrangements should be made to accommodate children at the workplace.

Mental health is one of the things that may be neglected during the epidemic situation, and maybe mental health is not considered as much as other issues such as taking care of the physical condition of the patients, changes in the physical environment, education related to the disease, etc. Communicating, regularly, and accurately with workers, providing information and answering questions, being available, limiting the use of media and social media, caring leadership culture, and equipping line managers with the necessary skills and tools to review how employees are working. Knowing the symptoms of distress can be a solution to maintaining mental health in an epidemic situation.

Studies have shown the effectiveness of closing schools and workplaces in reducing COVID-19 [50]. The pandemic of COVID-19 is associated with other occupational health and safety risks, such as ergonomic problems, work pressure and long working hours, remote working, psycho-social risks, poisoning, etc. Some chemicals used for disinfection may increase the risk of respiratory diseases [51]. Regarding psycho-social risks, we can mention the weight of responsibility, intellectual effort, multi-tasking, stress, job insecurity, and financial stress. With the outbreak of the COVID-19 pandemic, the workload increased, this increase and the perception of performance/efficiency is associated with the perception of greater demand due to remote working and compromise with household, family, and emotional duties in the management of the epidemic [52, 53]. Another phenomenon that has emerged parallel to this pandemic is the infodemic, an overabundance of information and not all of it being accurate and correct, making it difficult to find reliable and valid guidance for informed decision-making. Also, this issue causes discomfort and increases the risk of mental health disorders such as depression and anxiety [54]. Inadequate training, incorrect use of personal protective equipment, and non-compliance with protocols lead to self-contamination and transmission of infectious diseases [55]. Low awareness about how to use disinfectants [56] and incorrect and unsafe use of these disinfectants can lead to other toxic effects in people, which can be much more dangerous than the virus itself [57, 58].

The current COVID-19 pandemic requires sustainable behavior change to reduce the impact of the virus. Training on how to use technologies and access to relevant facilities, providing equipment for personal protection and disinfectants, providing information and instructions related to disease, and creating a safe work environment are among the responsibilities and duties of employers and managers that need to be considered [30, 59]. Studies have shown the effectiveness of public information campaigns in reducing COVID-19 [50].

Therefore basic, comprehensive, timely, and relevant training is one of the important issues that should be taken into consideration in pandemic conditions. Employees need to know how to act and need to be aware of relevant programs and processes.

Legal requirements and duties of employees and employers are other things that should be considered in pandemic conditions such as surveillance.

In this scenario, the health and safety of employees should take precedence. Working hours should adhere to established rules and agreements, while work pressure should be kept at a minimum [60]. As per the regulations of OSHA, employers are obligated to provide a work environment free of known hazards that could result in death or serious injury to their employees [61]. Employers can play an important role in better managing the impact of the pandemic on their employees by designing measures in various fields including work environment, workload, leadership and communication methods, work safety, and psychological and social support [52].

Adherence to infection control measures, particularly the appropriate utilization of personal protective equipment (PPE) and the practice of courting, when closely supervised, can potentially diminish the occurrence of weekly infections and mortality rates. Therefore, the monitoring of the implementation of the instructions and their compliance should be taken into consideration in the pandemic situation and there should be accountability from the employees and employers.

The COVID-19 pandemic is a challenge for public health and occupational medicine, and the design of prevention and protection strategies requires the expertise of many disciplines. For prevention, people must be motivated to participate, and this motivation depends on understanding how and why prevention measures are implemented [62]. Therefore, it is essential to engage in consultation and collaboration with stakeholders when making decisions related to risk assessment, reopening or suspending workplaces, reducing work activities, and implementing other measures for disease prevention and control in the workplace. Policies and decisions made with input and participation from all relevant parties should be adopted [48, 63].

Pandemic control requires having a comprehensive view and considering all related issues, so consulting and using different expertise in all stages should be considered. Also, cooperation between management and employees and their representatives should be an essential element of relevant workplace prevention measures.

Based on the results of the study, employers must develop and implement policies and procedures for all phases of reopening that address prevention, monitoring, and response to any emergence or resurgence of COVID-19 in the workplace or community [32]. After quarantine, resuming work while avoiding a re-epidemic is a significant challenge [64, 65]. Therefore, the reopening of workplaces should be carefully planned, taking into consideration all potential risks to health and safety, and ensuring they are properly evaluated and controlled. Additionally, even if conditions upon returning to work seem low-risk, the importance of implementing preventive and protective measures should be continuously emphasized. Work activities should resume in a phased manner, giving priority to tasks that are essential for protecting the health and the economy while allowing tasks that can be effectively completed remotely to be the last [66].

Reducing the prevalence of disease after reopening workplaces and believing that the risk of disease transmission will decrease is one of the threats after reopening that can lead to the next wave of epidemics. After the reopening of workplaces, all precautionary points should be taken into account as before and according to the risk assessment.

The results of a longitudinal cohort study in Massachusetts nursing homes showed adherence to infection control measures, particularly the appropriate utilization of personal protective equipment when closely supervised, can potentially diminish the occurrence of weekly infections and mortality rates [67]. To improve adherence to preventive measures against COVID-19, it is essential to monitor compliance with protocols. Monitoring the implementation of protocols is crucial for ensuring compliance [68–70]. Therefore, regular monitoring of guideline implementation, laws, and regulations should be included as a key component in the planning of action for following protocols and controlling the spread of the disease.

The documentation included in this study was limited to international guidelines available at the time of the study. Analyzing the content of national best practices and studies can help improve the findings of this study.

## Conclusion

According to the findings, the measures most likely to be neglected during the pandemic are mental health and financial support for businesses. This is because, at the beginning of every epidemic, the focus is on identifying and caring for sick and suspicious individuals, as well as implementing changes in the physical environment, hygiene, and personal protective measures. However, mental health and financial support are often overlooked due to their less visible nature and the lack of government funds and credibility. Another challenge faced was the lack of infrastructure for virtual and remote work, as well as the necessary skills, which the COVID-19 pandemic highlighted.

Protecting employees during a pandemic requires a multifaceted approach, including the adoption and enforcement of occupational health and safety laws and regulations, as well as strong advocacy. The action plan for pandemic control must be based on the level of risk, with support tailored to employees’ circumstances and needs. Controlling a hazard like a pandemic is best done systematically, often requiring a combination of control measures to ensure employee safety. Alongside implementing communication programs to address employee concerns and provide necessary training, it is crucial to consider the appropriateness and availability of communication channels, as well as the need for timely and understandable training for employees.

Cooperation among international organizations is essential to develop a standardized plan and issue comprehensive guidelines in response to health emergencies with a global perspective and local implementation, drawing from the lessons learned during the COVID-19 pandemic.

## Data Availability

All data generated or analyzed during this study are included in this published article.

## Abbreviations

CDC: Centers for Disease Control and Prevention
COVID-19: Coronavirus disease 2019
EU.OSHA: European Agency for Safety and Health at Work
ILO: International Labor Organization
NIOSH: National Institute for Occupational Safety and Health
OSHA: Occupational Safety and Health Administration
PPE: Personal Protective Equipment
WHO: World Health Organization
